# A scoping review of remote facilitation during simulation-based healthcare education

**DOI:** 10.1186/s12909-023-04551-3

**Published:** 2023-08-21

**Authors:** Ju Ok Park, Jannet Lee-Jayaram, Eri Sato, Yuka Eto, Melissa Kahili-Heede, Krystal Hirayama, Benjamin W Berg

**Affiliations:** 1 Department of Emergency Medicine, Hallym University College of Medicine, Dongtan Sacred Heart Hospital, Hwaseong-si, South Korea; 2 SimTiki Simulation Center, John A. Burns School of Medicine, University of Hawaii at Manoa, , Honolulu Hawaii, USA; 3 Health Science Library, John A. Burns School of Medicine, University of Hawaii at Manoa, Honolulu Hawaii, USA

## Abstract

**Background:**

Remote facilitation is a synchronous distance education method where instructors facilitate a lesson, in real-time, in physically separate conditions. In this scoping review, we aimed to describe types of remote facilitation used in a healthcare simulation, the influences on learner outcomes, and related factors.

**Methods:**

We accessed PubMed, EMBASE, CINAHL, ERIC, and Web of Science using our search strategies. Five reviewers performed the review using the Preferred Reporting Items for Systematic Reviews and Meta Analysis extension for Scoping Reviews (PRISMA-ScR) framework, and the Johanna Briggs Institute (JBI) guidelines.

**Results:**

We included a total of 29 articles presenting 28 simulation studies. The most common tool was videoconferencing (n = 26, 89.7%). Knowledge improvement was the most frequently measured outcome. There was no significant difference in learning outcomes between the two teaching modes. There were differences in learners’ preferences and satisfaction with remote facilitators before and after COVID-19.

**Conclusions:**

Our scoping review indicates that remote facilitation has been widely accepted in many healthcare professions using various types of simulation modalities. Remote facilitation can be used to overcome logistical problems of synchronous multi-location education, and to improve learner knowledge, skills, and confidence measured by instructor evaluation or self-assessment.

**Supplementary Information:**

The online version contains supplementary material available at 10.1186/s12909-023-04551-3.

## Introduction

During the COVID-19 pandemic, much of healthcare education, including simulations-based trainings, rapidly transitioned to remote platforms rather than in-person classes [1, 2]. However, remote simulation or telesimulation is not a new concept. Telesimulation is a process by which telecommunication and telesimulation resources are used to provide education, training, and/or assessment to learners at an off-site location [3]. Remotely facilitated simulation-based training (RF-SBT) is a synchronous distance education method in which instructors facilitate lessons in real time under physically separate conditions [4]. RF-SBT can be conducted using a variety of techniques. For example, trainees may have access to both patient simulators (e.g., a manikin, a standardized patient) and instructors remotely. In another approach, the simulators are on-site, but the instructors control and facilitate the lesson remotely with or without on-site instructors. In the many years since virtual technology for education was introduced, RF-SBT was initially be conducted with a screen-based simulation and has evolved to an application using other methods. The unifying key characteristic of these simulations is remote facilitation.

Several studies have reported that remote facilitation is effective and applicable in healthcare simulation education [5–10]. However, the perception of remote facilitation and its effect on educational outcomes are unclear. Several studies have reported learner satisfaction with remote facilitation [11–14], whereas other participants have indicated difficulties using technology, discomfort, and communication barriers [4, 7, 8, 10]. Before COVID-19, remote facilitation was used for specific reasons, such as education in underdeveloped countries, rural or resource-limited areas, or military medical practice [15–19]. Today, remote education is frequently considered in various healthcare education contexts because remote educational experiences using telecommunications technology have become common during COVID-19 [2]. Debriefing is essential for learning in all simulations, including virtual simulation experiences [20]. The question now is how best to conduct debriefings and facilitation during remote simulations, as educational institutions increasingly use online and technology-enabled learning [11]. In a recent meta-analysis, approximately half of the virtual simulations, regardless of whether they were operated remotely or locally, did not have post-simulation debriefing sessions [21]. Thus, most studies did not examine the effectiveness of debriefing sessions on student learning.

With this scoping review, we aimed to describe the types of remote facilitation in a healthcare simulation, the influence on learner outcomes, and other related factors. To achieve this goal, we scrutinized the circumstances and types of simulation education using remote facilitation, outcomes observed in simulation-based education with remote facilitation, learners’ preferences, and factors related to the effect of remote facilitation on learning outcomes.

## Methods

We followed the Preferred Reporting Items for Systematic Reviews and Meta Analysis extension for Scoping Reviews (PRISMA-ScR) framework [22] and the Johanna Briggs Institute (JBI) guidelines [23, 24].

### Inclusion criteria

To be included in the review, articles were required to describe simulation education and report information on facilitation and learning outcomes. We included peer-reviewed articles if they were published before April 2021 and written in English. We only included studies on remote facilitation conducted synchronously with simulation or consecutively conducted as a debriefing session by a facilitator with greater expertise than learners. We included all simulation topics and techniques, such as mannequins, task trainers, standardized patients, simulated patients, virtual reality and others in simulation education research in order to encompass all element of remote simulation facilitation to the fullest extent possible.

### Search strategy and screening

For this scoping review, we accessed PubMed, EMBASE, CINAHL, ERIC, and Web of Science using our search strategies (Supplementary material 1). We did not identify or include gray literature sources. We performed a systematic search of these databases from April 1 to May 13, 2021. The search strategies were developed by an experienced research librarian (MK) and further refined through reviewer discussions to identify relevant primary studies. The final search strategy for all databases is in Supplementary material 1. The final search outcome was deduplicated by the librarian (MK) following Bramer’s methods [25]. We included quantitative, qualitative, and mixed methods studies to understand the full scope of learning outcomes and the learners’ experiences. We excluded gray literature such as conference abstracts, editorials, interviews, lectures, letters, news, guidelines, methodology papers, and non-systematic reviews.

To clarify the inclusion criteria and increase consistency among reviewers, all reviewers conducted pilot screening using 50 papers randomly selected from the search results. During the pilot screening, the reviewers narrowed the inclusion criteria and revised the data extraction form.

**Fig. 1:**
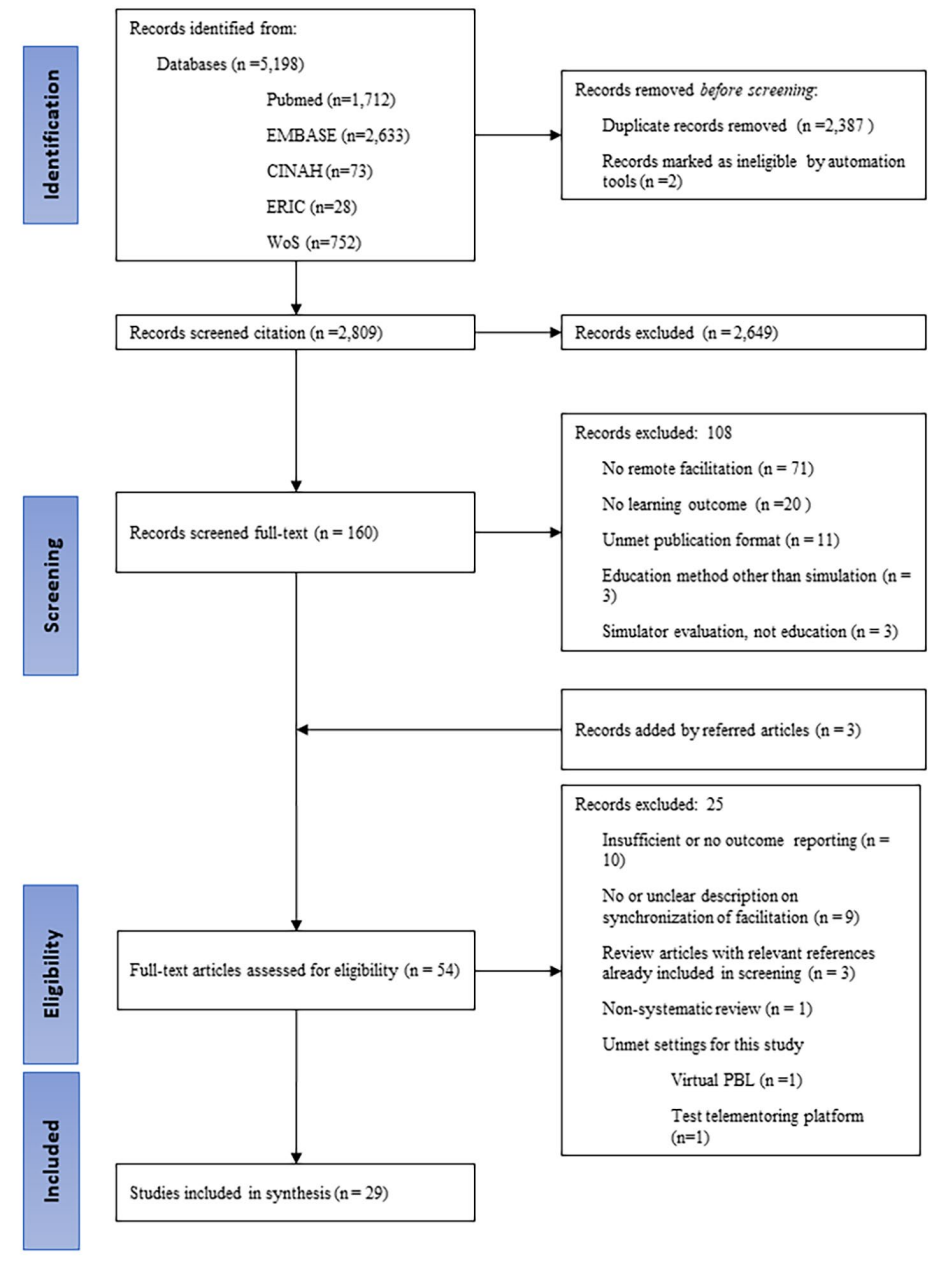
The Preferred Reporting Items for Systematic Reviews and Meta Analysis extension for Scoping Reviews (PRISMA-ScR) flow diagram of this scoping review

Four reviewers (BB, JL, YE, ES) working in pairs sequentially evaluated the titles, abstracts, and then the full texts of all articles. Each reviewer pair was responsible for half of the entire search results. The fifth author (JP) independently screened all findings. Thus, each paper was screened by three independent reviewers and decisions were made through the consensus of at least two reviewers. If the fifth reviewer determined that further discussion was necessary for an inclusion decision, all five reviewers reviewed and discussed the article to make a final decision (Fig. 1). Reviewers completed this process using the Colandr [26], a web-based evidence review platform.

### Data extraction and synthesis

A data extraction chart was primarily developed by one reviewer (JP) and three reviewers (BB, JL, JP) independently charted the data, discussed the results, and updated the data-charting format through an iterative process. Appraisal of the included articles was completed by four reviewers in two scoring groups of two members each using the Joanna Briggs Critical Appraisal Tools [27, 28]. Each scoring group was assigned and scored half of the total number of manuscripts. Individual reviewers within a scoring group reconciled discrepancies within the scoring group through discussion. Recent developments in the subject matter include the difficulty of applying a rigorous research design; we did not exclude studies based on appraisal scores.

Due to the heterogeneity of study designs and interventions, we synthesized, summarized, and reported on the extracted data in a descriptive format. We initially summarized the characteristics of simulation and facilitation, and summarized logistical and organizational, demographic, and other educational characteristics of simulation and facilitation. Next, we synthesized essential qualitative data through discussions based on the research question and themes related to learner outcomes. Finally, we narratively described the essential qualitative data through reviewer discussions based on the research question.

## Results

The initial systematic search of databases produced 5,198 records. After deduplication, the citation screening began with 2,809 articles and based on review titles and abstracts, we excluded 2,649 studies and downloaded 160 full-text manuscripts for further screening. After full-text screening, 54 articles were eligible for full-text review. Finally, we included 29 articles presenting 28 simulation studies in the scoping review (Fig. 1).

### Study characteristics

Two articles [4, 5] were from one simulation study. All eligible studies were published after 2009, and eight studies described simulations during the COVID-19 pandemic (Table 1). There was one qualitative study [4]. Among others, seven articles used a mixed methods design, including a quantitative design as a major approach with qualitative data collection such as a focus group interview [14, 29], survey comments [7, 30–32], and audiovisual recorded comments [33]. Reviewers evaluated the quality of these mixed methods articles using a JBI checklist for quantitative designs if the primary technique was quantitative. Most articles showed a medium (0.5–0.7) quality of evidence based on the appraisal score (Table 1).

**Table 1:** Characteristics of simulation in the included studies

Author (Year)	Implementation year of simulation	Population size	Target population	Simulation topic	Simulation techniques	Mean JBI appraisal score by two reviewers
Altieri (2020)	Pre-COVID19	65	Surgical residents	Electrosurgery	Task trainer, computer screen-based simulation	0.58
Burckett-St. Laurent (2016)	Pre-COVID19	19	Anesthetists	Ultrasound-guided regional anesthesia (UGRA) training	Task trainer	0.67
Cetrone (2020)	Pre-COVID19	40	Fourth-year medical students	Answering pages from the registered nurse (RN)	Simulated human (standardized RN)	0.50
Christensen (2015)	Pre-COVID19	305	Newly (within the first 12 months) graduated nurses or doctors	Management of the deteriorating patient	Computer enhanced manikins (Laerdal SimMan), simulated patients	0.65
Christensen (2018)	Pre-COVID19	157	Newly (within the first 12 months) graduated nurses or doctors	Management of the deteriorating patient	Computer enhanced manikins (Laerdal SimMan), simulated patients	0.95
Ciullo (2018)	Pre-COVID19	33	Third-year and fourth-year medical students from two medical schools	Mechanical ventilation training	Computer enhanced manikins	0.65
Hayden (2012)	Pre-COVID19	44	First- and second-year medical students	Performing a history and physical, ordering diagnostics, and performing interventions for patients with rhabdomyolysis, renal colic, anaphylaxis, pulmonary embolism, subdural hematoma, and septic shock.	Computer enhanced manikins	0.45
Heidemann (2020)	Pre-COVID19	27	Fourth-year medical students	Diagnostics, management, and communication items in acute gastrointestinal hemorrhage, atrial fibrillation with rapid ventricular response, septic shock, altered mental status, and severe hyperkalemia	Simulated human (Standardized RN)	0.50
Jewer (2019)	Pre-COVID19	69	First- and second-year medical students	Doing a chest tube insertion	Task trainer	0.54
Katz (2017)	Pre-COVID19	32	Ultrasound physicians and technicians	Ultrasonography imagery interpretation	Computer screen-based simulation (Wizard of OZ)	0.63
LaMarra (2020)	Pre-COVID19	79	Research coordinator	Getting informed consent in pediatric critical care research	Simulated patients	0.71
McCoy (2017)	Pre-COVID19	32	Fourth-year medical students	Management of critically ill patients	Observing a live simulation using computer enhanced manikins	0.62
Mikrogianakis (2011)	Pre-COVID19	22	Physicians in pediatrics, emergency medicine, surgery, and anesthesia	IO insertion techniques	Task trainer	0.75
Ohta (2017)	Pre-COVID19	176	Fifth-year medical students	Management of critical pediatric patients	Computer enhanced manikins (SimBaby)	0.64
Okrainec (2010)	Pre-COVID19	16	Fully trained surgeons and junior trainees.	Practice of laparoscopic skills	Task trainer	0.57
Phillips (2020)	Pre-COVID19	22	First-year nurse practitioner students	Telehealth	Simulated patients	0.72
Poland (2018)	Pre-COVID19	33	Third- and fourth- year medical students	Focused assessment with sonography for trauma (FAST) examination.	Task trainer (SonoMan)	0.60
Shershneva (2014)	Pre-COVID19	11	Family physicians, general internists, and family medicine residents	Motivational interviewing skills	Virtual reality (Second Life)	0.67
So (2019)	Pre-COVID19	26	Pediatrician, public health officials	Pediatric emergency preparedness (e.g., an outbreak of smallpox)	Virtual tabletop exercise	0.81
Verkuyl (2018)	Pre-COVID19	207	Fourth-year undergraduate nursing degree	Mental health and interpersonal violence assessment	Computer screen-based simulation, virtual reality (video game simulation)	0.70
Wen (2019)	Pre-COVID19	264	Students from the School of Medicine, Nursing, Pharmacy (remotely via videoconference), and graduate social work students	Interprofessional team simulation exercise involving a complex geriatric patient	Simulated patient and family	0.67
Kasai (2021)	Under COVID19	43	Fifth-year medical students	Writing medical records and interviewing	Computer screen-based simulation (Simulated electronic health record)	0.54
Loli (2021)	Under COVID19	40	General surgical resident	Laparoscopy	Task trainer	0.32
Mileder (2021)	Under COVID19	18	Medical students and neonatal nursing staff	Neonatal resuscitation training	Low-fidelity mannequin	0.44
Montgomery (2021)	Under COVID19	86	Emergency department RNs	Management of pediatric status epilepticus	Computer screen-based simulation (SimBox)	0.44
Patel (2020)	Under COVID19	53	Anesthesiology residents	Anesthetic management and resuscitation for pediatric intracranial epidural hematoma patient in operation room	Computer enhanced manikins (SimBaby)	0.83
Quaranto (2021)	Under COVID19	31	Third-year medical students	Basic surgical skills (knot tying, suture)	Task trainer	0.69
Tan (2021)	Under COVID19	69	Oral health therapy students (year 1, 2, 3)	Periodontal instrumentation, alginate mixing for dental impressions, rubber dam placement, dental charting, identification of dental instruments, soap carving using wax knives, and infection control training such as hand hygiene and donning of personal protective equipment	Task trainer	0.69
Yang (2021)	Under COVID19	48	Medical students (2nd, 3rd, 4th )	Pediatric acute care of a septic infant and a toddler with anaphylaxis.	Simulated human with computer screen-based simulation (Leardal LLEAP)	0.19

### Target population

The number of learners varied by type of simulation and the duration of the curriculum, from 11 [29] in an avatar-based virtual continuing medical education (CME) workshop to 305 [5] in a statewide mandatory course offered year-round. The most common areas of professional expertise among learners were medical [6, 7, 10, 14, 17–19, 29, 33–42], nursing [11, 31, 43], multi-professional [4, 5, 13, 44], interprofessional [30, 32], and dental [45]. One study [46] reported on an educational intervention for research coordinators. The most common learner levels were students [5–7, 10, 11, 14, 17, 31–33, 37, 38, 41, 42], while other learners were professionals who had completed professional school or other higher education and had practical experience, such as medical specialists [18, 19, 29, 30, 35], medical residents [34, 39, 40], nursing staff [43, 44], research coordinators [46], or post-graduate students [4, 5, 13].

### Simulation topics and modalities

Simulation topics for which remote facilitation was applied were very diverse. There were simulations for skills training [10, 13, 17, 18, 34, 35, 38, 39, 41, 44, 45], scenario-based simulations that included all or part of patient evaluation, diagnosis, and treatment [4–7, 11, 31, 37, 40, 42], In addition, there were simulations for interviewing patients or family members [29, 46], answering pages [33, 36], telehealth practice [31], medical records documentation [14], and interprofessional team training [30, 32]. Simulations focused on technical skills only [13, 17, 19, 34, 38, 39, 41, 47] (n = 8), non-technical skills (NTS), such as leadership, communication, teamwork, situation awareness and decision making, only [4–6, 10, 11, 30–33, 36, 40, 42, 46] (n = 13), and mixed (technical and non-technical) domains [7, 14, 18, 29, 35, 37, 44] (n = 8). Among NTS, communication was the most common domain.

With respect to the type of simulation modalities, simulations in the simulation center using only computer enhanced manikins [4–6, 10, 40, 42] or task trainers [17–19, 35, 38, 39, 41, 45] were common. One study, reported in two articles [4, 5], used a combination of mannequin simulators and simulated human patients. Simulations were primarily accomplished with learners participating in simulations in which equipment was installed in advance, such as a simulation center, captured on a camera, and observed synchronously by a facilitator in a remote location.

In some studies, simulated or standardized nurses were simulated via telephone [33, 36] or video [31, 32, 46]. Computer screen-based simulations, including virtual reality [11, 13, 14, 29, 30, 43], were used in many remotely facilitated simulations.

### Facilitation

Before COVID-19, most studies connected learners and facilitators in a different location in the same building or on the same campus. However, under COVID-19, seven of eight studies connected participants across multiple locations (more than 3) in which participant connected from a different site [14, 40–45] (Table 2).

**Table 2:** Summary of facilitation and program evaluations

Author (Year)	Number of connected sites	Remote facilitation technique	Remote demonstration by facilitator	Program evaluation (Kirkpatrick level)
Altieri (2020)	2	Videoconferencing (no comment)	No	2
Burckett-St. Laurent (2016)	2	Videoconferencing (Skype)	Yes	1, 2
Cetrone (2020)	2	Telephone	No	2
Christensen (2015)	2	Videoconferencing (Cisco Webex)	No	1, 2
Christensen (2018)	2	Videoconferencing (Cisco Webex)	No	1
Ciullo (2018)	2	Videoconferencing (FaceTime)	No	1, 2
Hayden (2012)	2	Videoconferencing (Cisco Webex)	No	1
Heidemann (2020)	2	Telephone	No	1, 2
Jewer (2019)	2	Videoconferencing (VSee)	Yes	1, 2
Katz (2017)	2	Web-based virtual platform (Wizard of OZ)	No	1, 2
LaMarra (2020)	≥ 3	Videoconferencing (Skype)	No	1, 2, 4
McCoy (2017)	2	Videoconferencing (Live TV internet connection)	No	1, 2
Mikrogianakis (2011)	2	Videoconferencing (Skype)	Yes	1, 2
Ohta (2017)	2	Videoconferencing (UltraVNC, Skype)	No	1, 2
Okrainec (2010)	2	Videoconferencing (Skype)	Yes	2
Phillips (2020)	2	Videoconferencing (no comment)	No	1, 2
Poland (2018)	2	Videoconferencing (FaceTime)	No	1, 2
Shershneva (2014)	≥ 3	Web-based platform (Second Life)	No	1, 2, 3
So (2019)	≥ 3	Videoconferencing (Zoom)	No	1, 2, 3, 4
Verkuyl (2018)	≥ 3	Videoconferencing (Zoom)	No	1, 2
Wen (2019)	2	Videoconferencing (no comment)	No	1, 2
Kasai (2021)	≥ 3	Videoconferencing (Zoom)	No	1, 2
Loli (2021)	2	Videoconferencing (WhatsApp )	No	1, 2
Mileder (2021)	≥ 3	Videoconferencing (Cisco Webex)	Yes	1, 2
Montgomery (2021)	≥ 3	Videoconferencing (Zoom)	No	2
Patel (2020)	≥ 3	Videoconferencing (Zoom)	Yes	1, 2
Quaranto (2021)	≥ 3	Videoconferencing (Zoom)	Yes	1, 2
Tan (2021)	≥ 3	Videoconferencing (Zoom)	Yes	1, 2
Yang (2021)	≥ 3	Videoconferencing (Zoom)	No	1, 2

The most common tool for remote facilitation was videoconferencing ([3–7, 10, 11, 14, 17–19, 30–32, 34, 35, 38–46]) (n = 26, 89.7%). Two studies [33, 36] used a telephone for immediate feedback after simulated paging education. One study [29] employed the verbal and synchronous chat function in Second Life® (https://secondlife.com/) for within-event facilitation. There was one study using a specified web-based platform, Wizard of OZ Telemedicine Simulator [12], including synchronous text feedback [13]. Via the videoconferencing system, the facilitator could demonstrate the technical skills enhancing teaching and debriefing in nine studies [17–19, 35, 40–42, 44, 45] (Table 2).

Facilitators’ backgrounds varied depending on the subject matter of the simulation, but most facilitators were expert clinicians or educators. Eight studies [5, 6, 11, 13, 19, 33, 36, 42] indicated that the facilitators were trained or certified for the simulation, and three of them reported that the facilitators were trained for remote facilitation [5, 11, 42]. Remote facilitation was conducted during the simulation in 7 studies [19, 29, 30, 34, 35, 39, 41], post-simulation [7, 11, 31–33, 36, 37, 42] in 8, or both [4–6, 10, 13, 14, 17, 18, 38, 40, 43–46] in 17.

### Learning outcomes of remotely facilitated simulations

Enhanced knowledge was the most commonly measured outcome. In most studies that compared facilitation methods, there were no significant differences in knowledge improvement between the on-site and remotely facilitated groups [5, 10, 11, 17, 34, 37]. Moreover, long term retention declined in both remotely and on-site facilitated groups [34]. One study using web-based immediate feedback improved knowledge in the remotely facilitated group more so than the group with no facilitation [13]. The pre- and post-studies [18, 30, 40, 44] for remote facilitation hint at improved knowledge as an outcome.

In the realm of technical skills, there were no significant differences in skills improvement between the on-site and remotely facilitated groups [17, 38]. In a simulation for focused assessment with sonography for trauma (FAST), the collective increase in knowledge was greater for the in-person group, whereas the improvement in FAST examination performance during the simulation was greater for the telementored group [38]. A remotely facilitated group improved their laparoscopic surgical technique more and reached a passing score more often than the self-practice group [19]. There was a statistically significant difference between on-site and remote facilitation in the change of clinical performance score to manage altered mental status to overdose, favoring on-site facilitation. In the same study, improvement in the management of dynamic hyperinflation with a mechanical ventilator revealed no difference between both types of facilitation [10]. In the pre- and post-studies, simulations with remote facilitation significantly enhanced in terms of writing medical records and summaries [14], in providing ultrasound-guided regional anesthesia [35], basic surgical skills (knot tying and suturing) [39], and neonatal resuscitation [44]. However, there was no change in the success rate of obtaining informed consent [46].

Other studies examined non-technical skills. When compared to on-site facilitation, remotely facilitated pediatric acute care teamwork training indicated no difference in performance between the groups [6]. In an interprofessional education (IPE) core competency simulation, there was statistically significant improvement in all scores when measured using pre-/post-testing [32].

Some studies have employed self-assessment by learners for program evaluation. In a study that compared remote and on-site facilitation, learners in both groups reported feeling greater comfort and competence for the simulation tasks after the course and 6 months later, without any difference between the groups [34]. Confidence was improved without a statistically significant difference between groups with various facilitation types in a FAST simulation [38], mechanical ventilation training [10], and the virtual gaming simulation of mental health assessment [11].

In an international remote facilitation study, learners reported significant improvement in their comfort, familiarity, and knowledge when inserting an intraosseous needle after simulation training [18]. Similarly, students’ aggregate confidence score rose significantly after interactive remote basic surgical skills during COVID-19 [41]. In addition, 90% of learners of pediatric patient care simulations agreed with the statements “I am more comfortable with pediatrics after this session” and “Participating improved my pediatric knowledge/skills” [42].

### Learners’ preferences and satisfaction

There were differences in learners’ preferences and satisfaction with remote facilitation before and after COVID-19. In studies conducted before COVID-19, either on-site facilitation was preferred [4, 5, 32] or there was no difference in the preference for type of facilitation [17, 37]. There were two studies that used the Debriefing Assessment for Simulation in Healthcare (DASH) for evaluation of debriefing, and DASH scores were significantly higher for the in-person facilitation group than the remote facilitation group [10, 38]. When comparing in-person, remote, and self-debriefing methods, in-person facilitation was most favored, followed by remote facilitation and finally self-debriefing [11].

There have been no studies comparing different types of facilitation under COVID-19. Student peer-teachers and neonatal nursing staff reported that they still preferred traditional face-to face instruction after a neonatal resuscitation training that connected the clinical skills center with students at home [44]. However, in a study that involved remote facilitation only, learners reported that remote facilitation achieved the intended learning objectives [46], was helpful for practice [36], and learner satisfaction levels were either acceptable [14] or high [45]. Furthermore, learners were likely to recommend remotely facilitated training to others with a high net promoter score [43]. Residents reported that remote facilitation seemed like a good educational tool [39] and could be a reasonable substitute for simulation with in-person facilitation [40].

### Barriers to, and enabling factors of, remote facilitation

Technical issues are among the most common barriers, such as low-speed internet in rural areas [17] or developing countries [19], and an unreliable internet connection, e.g., freezing, lost sound, or calls getting dropped [5, 10, 29, 31, 35, 40, 43, 44]. Moreover, audio-visual (AV) device operation issues— which interrupt learner engagement—have been reported, such as noise pollution from side conversations, echoes, and microphone feedback [40], as well as technical problems with AV devices in remote locations [32]. A few recent studies recommend checking for technical problems and providing an introduction to the connection modality or software in a pre-briefing session [41, 42]. In one study, the number of technical issues was greatest in the first session and declined over subsequent sessions [29]. In team simulations such as with IPE, it was difficult to gauge when the appropriate time was to speak using distance technology [32]. Headphones and high-definition cameras were utilized to overcome these technical constraints [44].

With respect to enabling factors of facilitation, a “time out (pause and reflect)” strategy and debriefing scripts were used to improve remote facilitation [42, 46]. A simulation coordinator, technician, assistant, or on-site expert helped with setup and troubleshoot any problem during remote facilitation [11, 36]. The facilitator and participants alike received training or orientation on remote technology [4, 5, 11, 30]. Modification via rehearsal or a pilot study prior to the actual simulation was helpful for remote facilitation [10, 11, 40].

## Discussion

In general, remote facilitation has been applied to diverse types of healthcare simulation trainings. The most common method of synchronous connection is through videoconferencing software or applications. Remote facilitation in simulation education consistently improves knowledge, skills, and confidence, measured either by instructor evaluation or self-assessment, and there was no significant difference from on-site facilitation or self-debriefing. Before COVID-19, students reported that they preferred on-site facilitation to remote facilitation, but after COVID-19, students became satisfied with remote facilitation and considered it a reasonable substitute for simulation with in-person learning. The barriers identified the most frequently were those related to communications technology, where prior orientation or training for remote technology could improve remote facilitation.

Remote or distance simulation with synchronous facilitation has been around for over a decade [48–50], connecting learners who are separated from their instructors, via communications technology, using various simulation modalities including human patient simulators, SPs, and task trainers. Given the long history of use, terminology or nomenclature requires definitions. Standardized terminology and reporting standards can clarify the understanding of outcomes, align methodology for research, and aid in the replication of educational curricula in numerous settings. In our scoping review, we found multiple terms and nomenclature referring to similar and dissimilar activities. The terms *telesimulation* [3, 17–19, 37, 40, 42–44, 46], *telementoring* [34, 39], and *telepresent* training [10, 38] were all used to describe similar and dissimilar simulation activities using audiovisual communications technology as a platform to connect instructors and learners in separate locations.

The simulation modalities varied within these and included human patient simulators, simple manikins, SPs, task trainers, actors, and video recordings. The term *virtual*, while used as a generally accepted reference to computer-based simulation [13, 29, 51], has also been applied to non-computer based, remote, or distance simulation [30, 39, 40]. Recognizing the need for organization and definitions, in 2020, the Society for Simulation in Healthcare published an addendum to the Healthcare Simulation Dictionary, 2nd edition [52] to include language for conducting simulations at a distance.

In recognizing that definitions specifically for virtual simulation have been variably reported, Cant et al. [53] proposed a common nomenclature to describe the modalities of computer-based simulation, specifying 3 categorical descriptions to include the level of fidelity, the presence of immersion or interactivity, and the form of the patient, whether a simulated patient or a computer-generated avatar. This proposal was limited to computer-based simulation and did not address instructor facilitation during interactive simulations. We suggest that a similarly expanded framework be considered to describe remote facilitation. This framework could include (1) a description of facilitation timing (synchronous or asynchronous); (2) a method for connecting learners and instructors (e.g., videoconferencing, telephone, computer-based); (3) a simulation modality and its location with respect to the participants (e.g., a human-patient simulator, simple manikin, task trainer, SP, actor, computer-based); and finally (4) a method of facilitation, feedback, and/or debriefing (e.g., via chat, verbally, or through visual feedback or demonstration, facilitated video discussion, computer-based via avatars, etc.).

A touted advantage of computer-based simulation is the ability to provide asynchronous guidance and feedback. There were not enough strictly computer-based simulations included in this scoping review [11, 13, 29] to allow comparison of computer-based to other simulation modalities, since our search specified synchronous facilitation activities. Given the increased experience with computer-based simulation and the expected expansion of distance learning, we anticipate growth in this area. More studies comparing computer-based simulation to other methods of remote simulation are needed. Computer-based simulation does not necessarily require active synchronous facilitation, nor does it mean that learners and instructors must be physically separated, although they may be.

Learner outcomes at Kirkpatrick levels 1 (reactions) and/or 2 (knowledge and skill acquisition) were noted in all reports. Level 2 was observed in 27, and level 1 in 25 reports. Level 3 and 4 outcomes were mentioned in 2 studies. Learners readily accepted and mentioned positive attitudes regarding remote facilitation, and knowledge outcomes improved uniformly using pre-/post-intervention assessments. Most studies did not make direct comparisons between remote and on-site in-person scenario facilitation and/or debriefing. The six studies that included comparisons showed no difference in knowledge outcomes or latency of knowledge decay. Kirkpatrick level 2 outcomes, regarding technical skills acquisition with remote facilitation, were demonstrably positive in reports on ultrasound and laparoscopic skills. However, direct comparisons were not uniformly equivalent, with some cases exhibiting better learning outcomes through on-site facilitation. These limited findings suggest that remote facilitation, when employed using optimum techniques, may yield equivalent knowledge outcomes, warranting further study of outcomes and determination of best practices with respect to remote facilitation instructional techniques, including immediate versus delayed individualized feedback, group debriefing formats, scenario facilitation approaches, and the selection of learning objectives (e.g., technical skills and/or knowledge).

Remote facilitation was conducted prior to COVID-19 in 21 reports and in 8 since COVID-19 restrictions in many healthcare education programs resulted in the transition of varying proportions of traditional curricula to distance and remote learning strategies. Prior to COVID-19, learners preferred on-site facilitation and scored on-site debriefers more highly than remote debriefers. More positive learner and facilitator level 1 reactions, including preferences, equivalency, and net promotor scores for remote facilitation in reports of learner experiences, were reported following the onset of the COVID-19 pandemic The positive reactions toward remote facilitation in the COVID era, while not directly compared to remote facilitation outcomes in the pre-COVID era, imply that deepening one’s familiarity with remote educational experiences and the adaptation of personal self-learning strategies may contribute to acceptance and effectiveness of novel approaches to distance learning, including remote facilitation of simulation-based healthcare education. On the other hand, in one survey administered to any profession involved in healthcare simulation in 2021, 67% of respondents considered the involvement of learners in distance simulation to be more challenging than in-person simulation. In particular, 52% of respondents believed that distance simulation made it more difficult to achieve learning objectives, but 40% thought they were similar to individual simulations [54]. To succeed in remote facilitated simulation education, both learners and instructors should be prepared and trained in techniques for this new trend of education.

This study contains a systematic, detailed review of the literature available for remote facilitation. We chose the scoping review method to derive a broader range of results and criteria since there was a lack of predefined criteria for remote facilitation and related learning outcomes. Gray literature, including editorials, interviews, news, non-systematic reviews, and non-peer reviewed literature were excluded due to lack of robust source verification and/or sufficient detail regarding simulation techniques or facilitation methods to meet the evaluation criteria of this scoping review. However, the publications of simulation studies reported using diverse study types resulted in a significant heterogeneity of studies, posing a challenge for data comparison. Finally, many simulation education studies did not adequately report the types and methods of facilitation for inclusion in this scoping review. For future research, the development of standardized reporting guidelines for simulation education and facilitation is essential [55].

The COVID-19 pandemic persisted and lasted longer than expected, and simulation centers have gradually transformed to meet the circumstances of the COVID-19 pandemic. In our search, we targeted studies released before April 2021, and the COVID-19 pandemic continues so far. In this context, more studies on remote facilitation may have been published after our search. Because we wanted to investigate the nature of remote facilitation and set the scope for it (versus a response technology in a particular situation such as COVID-19), it was enough to investigate the research of the above period.

This study offers insight into remote facilitation in healthcare simulations. Our scoping review indicates that remote facilitation has been widely accepted across many healthcare professions and various kinds of simulation modalities. Remote facilitation has been used to overcome logistical problems in terms of synchronous, multi-location education, as well as to improve knowledge, skills, and confidence measured by instructor evaluation or self-assessment. As learners have grown more accustomed to remote facilitation amidst COVID-19 and their preference for it has increased, new education through high-quality remote facilitation can be considered and adapted in a changing educational context.

### Electronic supplementary material

Below is the link to the electronic supplementary material.

Supplementary Material 1

## Data Availability

The code of database search during this study are included in the supplementary material.
