# Supplementary material for: A scoping review of remote facilitation during simulation-based healthcare education
Source: BMC Med Educ. 2023 Aug 21;23:592. doi: 10.1186/s12909-023-04551-3 (PMC10464104; doi:10.1186/s12909-023-04551-3)
Supplement: Supplementary file 1 — Supplementary Material 1 [file 12909_2023_4551_MOESM1_ESM.docx]

Supplementary material 1. The systematic search strategies for each database

| DB | Search | Results |
| --- | --- | --- |
| PubMed | ((((debrief* OR feedback OR facilitat*)) AND ((remot* OR virtua*) OR (tele*) OR (distanc*) OR (synchronous))) AND ((Simulation training) OR (Simulation education))) OR (telesimulation) Filters: from 1000/1/1 - 2021/3/31 | 1,712 |
| EMBASE | ((simulation AND training:ab,ti,kw,de) OR (simulation AND education:ab,ti,kw,de) OR telesimulation:ab,ti,kw,de) AND ((debrief*:ab,ti,kw,de OR feedback:ab,kw,ti,de OR facilitat*:ab,ti,kw,de) AND (tele*:ab,kw,ti,de OR distanc*:ab,kw,ti,de OR synchronous:ab,kw,ti,de OR (remot*:ab,kw,ti,de OR virtua*:ab,kw,ti,de))) | 2,633 |
| CINAHL | ((((debrief* OR feedback OR facilitat*)) AND ((remot* OR virtua*) OR (tele*) OR (distanc*) OR (synchronous))) AND ((Simulation training) OR (Simulation education))) OR (telesimulation) | 73 |
| ERIC | (MAINSUBJECT.EXACT.EXPLODE("Graduate Medical Education") OR MAINSUBJECT.EXACT.EXPLODE("Medical Education")) AND (((((debrief* OR feedback OR facilitat*)) AND ((remot* OR virtua*) OR (tele*) OR (distanc*) OR (synchronous))) AND ((Simulation training) OR (Simulation education))) OR (telesimulation) ) | 28 |
| Web of Science | ((((debrief* OR feedback OR facilitat*)) AND ((remot* OR virtua*) OR (tele*) OR (distanc*) OR (synchronous))) AND ((Simulation training) OR (Simulation education))) OR (telesimulation) | 752 |
